# Synergistic action between extracellular products from white-rot fungus and cellulase significantly improves enzymatic hydrolysis

**DOI:** 10.1080/21655979.2017.1308991

**Published:** 2017-04-28

**Authors:** Yushan Wang, Yang Shao, Xinyue Zou, Mandi Yang, Lin Guo

**Affiliations:** Department of Biotechnology, Heilongjiang Vocational Institute of Ecological Engineering, Heilongjiang Province, China

**Keywords:** cellulase, extracellular products, non-protein substances, synergistic action, white-rot fungus

## Abstract

With a set of perfect extracellular lignocellulolytic enzymes, white-rot fungus has been recognized as playing an important role in the degradation of lignocellulose materials, which leads to the possibility of creating a composite enzymatic system with high hydrolysis efficiency *in vitro. Echinodontium taxodii* is a promising white-rot fungus for biologic pretreatment. In this study, we extracted the extracellular products of *E. taxodii* under solid-state fermentation conditions, mixed the extracellular products with cellulase to build a composite enzymatic system, and systematically evaluated the effect of this system on the hydrolysis of acid-pretreated and raw maize stovers. We found that the extracellular products from *E. taxodii* could significantly improve the hydrolysis efficiency of cellulase, with a synergistic action between the extracellular products and cellulase. Corn stovers treated with extracellular products were suitable for the enzymatic hydrolysis of cellulase. Furthermore, we found that pure proteins from the extracellular products were not sufficient to generate synergistic action. This finding suggests that non-protein substances may also be involved in the synergistic action between the extracellular products and cellulase.

## Introduction

Low-cost and abundant agricultural residues, such as maize stovers and rice straw, are recognized as ideal sources of fermentable sugars for biorefining on a sufficiently large scale.^1,2^ Except for the small amount of straw used for paper and textiles, these residues either accumulate in the environment or are burned on-site, leading to severe environmental pollution and resource waste. Therefore, efficient utilization of lignocellulose materials, such as crop stovers, is important for sustainable agricultural development and protection of the ecological environment. Biorefining, based on sugar conversion, is an effective way to utilize resources that are otherwise wasted.^3,4^ However, due to the physicochemical properties and supramolecular architecture of lignocellulose materials, sugar conversion is greatly limited,^5,6^ and pretreatment is required for sugar release.^7-9^ Studies have shown that thermo-chemical pretreatment can reduce the conversion resistance of lignocellulose materials by reducing cellulose crystallinity and the content of lignin and hemicellulose in crop straw, thereby improving enzymatic hydrolysis.^5,10-13^ Commonly used thermo-chemical pretreatment methods often use strong chemicals and/or high temperatures and require large amounts of water.^14,15^ In addition, residual chemicals and inhibitory factors cause secondary pollution due to poor post-treatment practices.^16,17^

Biological pretreatment has been reported as a safe and environmentally friendly method for lignin removal from lignocellulose.^18-21^ The most promising microorganism for biologic pretreatment is white-rot fungus.^19,22-24^ Lee *et al.* found that the white-rot fungus *Stereum hirsutum* selectively degrades the lignin component of softwood rather than the holocellulose component. After pretreatment with *S. hirsutum*, it was found that the sugar yield increased greatly compared with non-pretreated control samples, reaching up to 21%.^21^ The pretreatment of lignocellulose materials with white-rot fungus primarily relies on its extracellular products, such as cellulase, hemicellulase, laccases, manganese peroxidases, etc.^25-27^ Together, these enzymes cause the degradation of lignocellulose materials.^21,28^ This fact suggests the possibility of realizing the enzymatic hydrolysis of lignocellulose materials using cellulase and the extracellular products of white-rot fungi. Additionally, synergistic action among the extracellular products has been found, significantly improving the degradation of lignocellulose materials. Varnai *et al.* confirmed that the supplementation of pure cellulase with non-specific endoglucanase and xylanase enhances the hydrolysis of substrates by more than 50%. When used together, xylanolytic and mannanolytic enzymes act synergistically with cellulase and improve the hydrolysis of lignocellulose materials.^29^ The question of how extracellular products from white-rot fungus interact with cellulase during degradation has received much interest.

*E. taxodii* is used for the biologic pretreatment of lignocellulose materials and has a strong ability to degrade lignin.^30,31^ In this study, we aimed to build a new kind of composite enzymatic system using cellulase and extracellular products from *E. taxodii*. Because different extracellular products exert different effects on the enzymatic hydrolysis of different substrates, we chose raw and acid-pretreated maize stovers as substrates. We systematically evaluated the effect of the composite enzymatic system on enzymatic hydrolysis, explored the interactive mechanism of extracellular products and cellulase, and found new factors affecting enzymatic hydrolysis.

## Results and discussion

### A composite enzymatic system containing the extracellular products of *E. taxodii* and cellulase can improve the efficiency of maize stover hydrolysis

We extracted the extracellular products of *E. taxodii* cultured in a solid-state fermentation medium, built a new kind of composite enzymatic system by mixing the extracellular products with cellulase, and obtained activated cellulase in solution at concentrations up to 50 FPU/ml. Then, we systematically evaluated the effect of the composite enzymatic system on the hydrolysis of raw and acid-pretreated maize stovers. First, we measured the activity of lignin degradation enzymes and cellulase in the extracellular products of *E. taxodii* under the conditions of solid-state fermentation (Table 1). The results showed a low activity of cellulase in the extracellular products of *E. taxodii*. However, laccase activity was easily detected under solid-state fermentation conditions. At 5 d of solid-state fermentation, laccase activity in the extracellular products reached a maximum; after 5 days, the activity gradually decreased with incubation time. Laccase activity reached up to 3653.33 IU/ml. Manganese peroxidases were also found in the extracellular products of *E. taxodii*. As the incubation time lengthened, the activity of manganese peroxidases decreased after an initial increase. The activity of manganese peroxidases in the extracellular products reached a maximum at 10 days, at 225.63 IU/ml. This finding indicates that these enzymes may contribute to the degradation of lignocellulose materials. This result also demonstrates the possibility of building a composite enzymatic system using extracellular products and cellulase.

**Table 1.** Activity of lignocellulolytic enzymes in solution with extracellular products.

	5 days	10 days	15 days
Cellulase (FPU/mL)	0.08 ± 0.01	0.12 ± 0.01	0.16 ± 0.02
Laccase (IU/L)	3653.33 ± 2.56	1008.35 ± 1.23	358.79 ± 4.56
Manganese Peroxidase (IU/L)	135.10 ± 1.55	225.63 ± 4.87	0.00 ± 0.00

*E. taxodii* was maintained in a solid-state fermentation medium at 28°C. The extracellular products were extracted after continuous culture for 5, 10 and 15 days, and the activity of lignocellulolytic enzymes was measured. The values are expressed as the mean ± SD of biologic triplicates (n = 3) representing 3 independent experiments.

**Figure 1.**
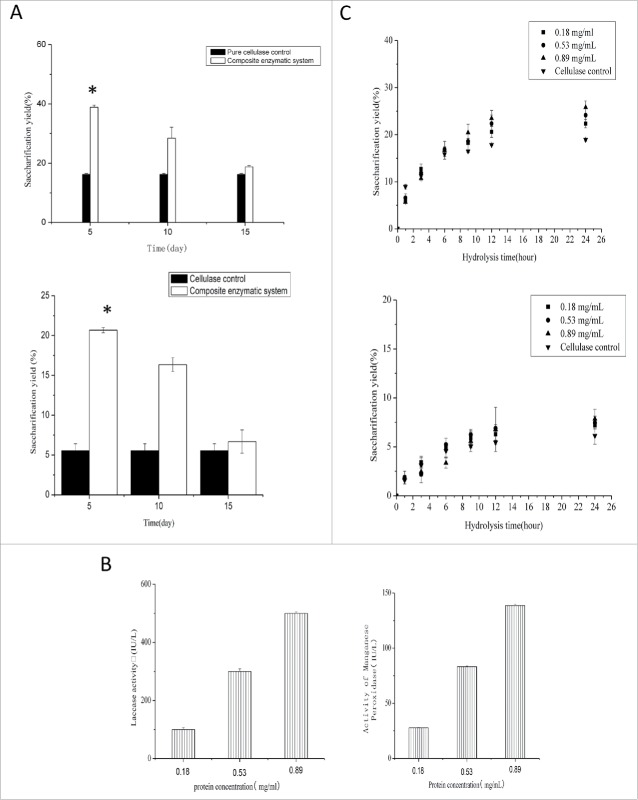
Effect of the composite enzymatic system on enzymatic hydrolysis. (A) The composite enzymatic system was created by mixing extracellular products with cellulase. First, 2.5% maize stovers were added to the composite enzymatic system and incubated at 45°C for 24 hours (top, acid-pretreated maize stover; bottom, raw maize stover). The results are expressed as the mean percent (mean ± SD of 3 independent experiments). *P<0.05 represents a significant difference compared with the pure cellulase control. (B) Extracellular proteins were extracted, and enzymatic lignin degradation was measured under different protein concentrations (0.18, 0.53 and 0.89 mg/ml) (left, laccase; right, manganese peroxidase). (C) Extracellular proteins were mixed with cellulase and subjected to enzymatic hydrolysis (top, acid-pretreated maize stover; bottom, raw maize stover).

It has been shown that the efficiency of enzymatic hydrolysis varies when unique extracellular products react with different substrates.^32^ In this paper, we used raw and acid-pretreated maize stovers as substrates and studied the effect of the composite enzymatic system on enzymatic hydrolysis. We found that the extracellular products at 5 d of solid-state fermentation can significantly improve enzymatic hydrolysis (Fig. 1A). At 24 hours of enzymatic hydrolysis, the saccharification yield of acid-pretreated maize stover reached 38.8%, a 1.4-fold increase over the cellulase control. For raw maize stover, the extracellular products also significantly improved the hydrolysis efficiency of cellulase. The saccharification yield of raw maize stover reached 20.68% after 24 hours of enzymatic hydrolysis, a 2.73-fold increase over the pure cellulase control. Thus, extracellular products at 5 d of solid-state fermentation were used to study the mechanism of enzymatic hydrolysis in subsequent experiments. We also found that the saccharification yield of the composite enzymatic system was close to that obtained for fungus treatment.^21^ Similar to fungus treatment, the composite enzymatic system may also be an effective tool for degrading maize stovers. However, unlike fungus treatment, no sugar is consumed when using the composite enzymatic system.

### Synergistic action occurs between the extracellular products and cellulase

Sequential enzymatic hydrolysis was performed before we studied the mechanism of the composite enzymatic system. We treated maize stovers with extracellular products of *E. taxodii* for 24 hours and then removed the extracellular products. Afterwards, the maize stovers were washed 3 times with distilled water and introduced into a reaction system of pure cellulase. The total saccharification yields of sequential enzymatic hydrolysis were calculated. For a control in the sequential enzymatic hydrolysis experiment, we replaced the extracellular products with distilled water. Table 2 shows that during the sequential enzymatic hydrolysis experiment, the pretreatment of maize stovers with extracellular products improved the hydrolysis efficiency of cellulase. The total saccharification yield of acid-pretreated and raw maize stovers reached 28.8% and 13.98%, respectively, indicating a 0.8- and 2.23-fold change, respectively, compared with the control. However, the saccharification yield of the sequential enzymatic hydrolysis was significantly lower than that of the composite enzymatic hydrolysis. This finding suggests that during hydrolysis with the composite enzymatic system, the extracellular products may synergize with cellulase, thus improving the hydrolysis efficiency of cellulase.

**Table 2.** Saccharification yield in each step of sequential enzymatic hydrolysis.

	Saccharification yield (%)		
	Treated with extracellular products	Hydrolysis of cellulase	Total
Acid-pretreated maize stover			
Extracellular products	0.45 ± 0.01	28.35 ± 0.54^**^	28.80 ± 0.33^**^
Water control	0.67 ± 0.10	15.33 ± 1.40	16.00 ± 0.11
Raw maize stover			
Extracellular products	0.52 ± 0.06	13.46 ± 0.05^**^	13.98 ± 0.10^**^
Water control	0.76 ± 0.01	3.57 ± 0.83	4.33 ± 0.57

Corn stovers were treated with extracellular products at 45°C for 24 hours, rinsed with distilled water, and then subjected to hydrolysis in a pure cellulase system. For a control, the extracellular products were replaced with distilled water. The results are expressed as the mean percent (mean ± SD of 3 independent experiments).

** P<0.01 compared with the water control.

### Synergistic action is derived from the interaction between the extracellular products and the substrate

In the composite enzymatic system, the synergistic action may arise from 2 factors: the extracellular products may increase the activity of cellulase, and the extracellular products may react with the substrates through the aid of degradation enzymes. First, we detected the changes over time in total cellulase activity in the composite enzymatic system during incubation (Table 3). The results showed that the cellulase activity remained constant, demonstrating that the synergistic action was not caused by changes in cellulase activity.

**Table 3.** Changes in cellulase activity as a function of incubation time.

Incubation time (hour)	Cellulase activity (FPU/g)
0	50.00 ± 0.3
6	49.56 ± 0.6
12	50.12 ± 0.6
24	49.85 ± 3.0

Extracellular products were mixed with cellulase to build a composite enzymatic system. As the incubation time increased, the total cellulase activity in the composite enzymatic system was measured. The values are expressed as the mean ± SD of biologic triplicates (n = 3) representing 3 independent experiments.

To study the effect of extracellular products on the enzymatic hydrolysis of maize stovers, we measured the saccharification yield of the maize stovers during sequential enzymatic hydrolysis after pretreatment with extracellular products. A pretreatment with distilled water was used as a control. Table 2 shows that after pretreatment with the extracellular products, the saccharification yields of raw and acid-pretreated maize stovers reached 13.46% and 28.35%, signifying a 2.77- and 0.85-fold change, respectively, compared with the control. Therefore, the synergistic action, which improved the hydrolysis activity of cellulase, resulted from the extracellular products reacting with the substrate.

### Non-protein substances also affect synergistic action

The extracellular products of white-rot fungi are very complex. A previous study demonstrated a high activity of laccase and manganese peroxidase in the extracellular products of *E. taxodii*. These enzymes play an important role in the degradation of lignin and may contribute to the generation of synergistic action. To determine the role of these enzymes, we extracted the proteins from the extracellular products and mixed them with cellulase. We then measured the effect of these proteins on the hydrolysis efficiency of cellulase. In a subsequent experiment, we used the protein concentration of the extracellular products (0.53 mg/ml) as a benchmark and selected other concentrations (0.18 and 0.89 mg/ml) around this benchmark. We found a high activity of laccase and manganese peroxidase in these proteins, which gradually increased with protein concentration (Fig. 1B). Furthermore, we observed the effect of different protein concentrations on the hydrolysis of cellulase. The results showed that the proteins from the extracellular products can improve the enzymatic hydrolysis of cellulase (Fig. 1C). However, the saccharification yield for only the protein complexes was lower than that for the composite enzymatic system, indicating that pure proteins from the extracellular products are not sufficient to generate the synergistic action. Thus, non-protein substrates from the extracellular products may also have an important role in generating synergistic action.

## Discussion

It is known that lignin degradation enzymes contribute to the degradation of lignin and improve saccharification yield.^21,27^ Due to their large molecular structure, these enzymes cannot easily permeate the microporous structure of lignocellulose. Thus, the degradation of lignin, particularly internal lignin, is greatly limited.^33^ In its growth process, white-rot fungi can produce some non-protein substrates, which play important roles in the degradation of lignin and the depolymerization of cellulose.^33,34^ Compared to enzymes, non-protein substrates may be able to more easily enter and react with the materials. Thus, these non-protein substrates may be involved in the improvement of hydrolysis efficiency.

In this study, we found *E. taxodii* to be a promising white-rot fungus for building a composite enzymatic system from its extracellular products and cellulase. The extracellular products at 5 d of solid-state fermentation can be used to build the composite enzyme system. We also found that the improved enzymatic hydrolysis was caused by synergistic action between the extracellular products and cellulase. The interaction between extracellular products and the maize stovers led to synergistic action with the cellulase and significantly improved the hydrolysis efficiency of cellulase. Pure proteins from the extracellular products were not sufficient to generate synergistic action with cellulase; non-protein substrates are also essential. This study revealed the relationship between extracellular products and cellulase in the degradation of maize stovers. We also presented a clean and effective method for obtaining reduced sugar from maize stovers, laying a foundation for the study of bioenergy and biofuel generation from lignocellulose materials.

## Materials and methods

### Microorganism and inoculum

*E. taxodii* was isolated (the forest of the Greater Khingan mountain, Heilongjiang, China) and maintained on potato dextrose agar (PDA) slants at 4°C. The inoculum was grown on potato dextrose broth (PDB) medium for 7 d at 28°C.

### Extraction of extracellular products

Corn stovers were ground, passed through a 0.9-mm screen, and then air-dried. Solid-state fermentation was performed in 500-ml Erlenmeyer flasks with 15 g of maize stalks and 10 ml of distilled water. The flasks were sterilized in an autoclave for 30 minutes at 121°C and aseptically inoculated with 10 ml of fungal inoculum. The cultures were statically incubated at 28°C, and the extracellular products were extracted after continuous culture for 5, 10 and 15 d. Distilled water (10 times the volume of the culture) was added to the flask, and the flask was rotated at 28°C for 4 hours. After filtration, extracellular products were separated from the residue, collected and used to build the composite enzymatic system. The activity of lignocellulolytic enzymes in the extracellular products was then measured.

### Extraction of extracellular proteins

Extracellular proteins were extracted from the extracellular products by precipitation with ammonium sulfate. The proteins were then freeze-dried into a powder under vacuum.

### Enzymatic hydrolysis

The composite enzymatic system was created by mixing the extracellular products with cellulase; the total activity of cellulase in the composite enzymatic system reached 50 filter paper units (FPU) per gram of maize stover. The enzymatic hydrolysis experiments were performed at 45°C for 24 hours using a 2.5% solution of maize stovers (w/v) in 8 ml of the composite enzymatic system. We replaced the composite enzymatic system with 50 mmol/l sodium acetate buffer (pH 4.8) and cellulase (50 FPU per gram of maize stover) as a control. The reaction was interrupted after hydrolysis, and the mixture was filtered through filter paper. The amount of reduced sugar after enzymatic hydrolysis was measured with 3,5-dinitrosalicylic acid (DNS). To study the enzymatic hydrolysis of the extracellular proteins, we mixed the proteins with cellulase. Three parallel hydrolysis experiments were performed at each pretreatment time using a commercial cellulase preparation (Wuxi Xuemei Enzyme Preparation Technology Co., Wuxi, China).

Sequential enzymatic hydrolysis was performed to observe the effect of pretreatment on enzymatic hydrolysis. First, 2.5% substrates were pretreated with the extracellular products at 45°C for 24 hours. After the supernatant was removed, pretreated maize stovers were rinsed 3 times with distilled water and were subjected to enzymatic hydrolysis with pure cellulase at 45°C for 24 hours.

The saccharification yield was calculated as follows:

$$Saccharification\;\;yield(\%)=\frac{Amount\;of\;glucose\;produced\;after\;enzymatic\;hydrolysis\times 0.9\times 100}{Amount\;of\;glucan\;in\;the\;corn\;stovers}$$

### Enzyme activities

The extracellular products were assayed to measure ligninolytic and hydrolytic enzyme activity. The activities of total cellulase and laccase were measured in 50 mmol/l sodium acetate buffer (pH 4.8) at 50°C by monitoring the release of reduced sugar. The amount of released sugar was measured using filter paper strips and ABTS as substrates.^35-37^ The manganese peroxidase activity was measured by monitoring the formation of Mn3+-malonate complexes at pH 4.5 in 50 mol/l sodium malonate buffer; one unit of enzyme activity was defined as the amount of enzyme required to release 1 μmol of product per minute under assay conditions.^36,38-40^

### Chemical analysis

The carbohydrate and lignin contents were determined based on “Determination of structural carbohydrates and lignin in biomass” from the National Renewable Energy Laboratory (NREL).^41^

### Data analysis

Mean values are presented with their standard errors. Multiple comparison tests were performed with Student's *t* test (significance levels = 0.05), and experimental results were validated by statistical analysis using Origin 8.0 software.
